# Human genetic admixture through the lens of population genomics

**DOI:** 10.1098/rstb.2020.0410

**Published:** 2022-06-06

**Authors:** Shyamalika Gopalan, Samuel Pattillo Smith, Katharine Korunes, Iman Hamid, Sohini Ramachandran, Amy Goldberg

**Affiliations:** ^1^ Department of Evolutionary Anthropology, Duke University, Durham, NC 27708, USA; ^2^ Center for Computational Molecular Biology, Brown University, Providence, RI 02912, USA; ^3^ Department of Ecology, Evolution and Organismal Biology, Brown University, Providence, RI 02912, USA; ^4^ Data Science Initiative, Brown University, Providence, RI 02912, USA

**Keywords:** admixture, genetic diversity, population genetics

## Abstract

Over the past 50 years, geneticists have made great strides in understanding how our species' evolutionary history gave rise to current patterns of human genetic diversity classically summarized by Lewontin in his 1972 paper, ‘The Apportionment of Human Diversity’. One evolutionary process that requires special attention in both population genetics and statistical genetics is admixture: gene flow between two or more previously separated source populations to form a new admixed population. The admixture process introduces ancestry-based structure into patterns of genetic variation within and between populations, which in turn influences the inference of demographic histories, identification of genetic targets of selection and prediction of complex traits. In this review, we outline some challenges for admixture population genetics, including limitations of applying methods designed for populations without recent admixture to the study of admixed populations. We highlight recent studies and methodological advances that aim to overcome such challenges, leveraging genomic signatures of admixture that occurred in the past tens of generations to gain insights into human history, natural selection and complex trait architecture.

This article is part of the theme issue ‘Celebrating 50 years since Lewontin's apportionment of human diversity’.

## 1. Introduction

In his foundational 1972 study ‘The Apportionment of Human Diversity’, Richard Lewontin demonstrated that the majority of human genetic diversity at a single locus is contained within, rather than between, populations using polymorphism data from a global sample [1]. The field continues to strive to understand the evolutionary processes that shape this important empirical observation. Notably, genomic data have revealed the extent to which one such process—genetic admixture—has been ubiquitous throughout human history and can shape the distribution of human genetic diversity in ways different from those predicted by classic population genetic models [2–5]. Here we focus on admixture as a population-level process, whereby gene flow occurs between previously diverged source populations, producing new populations with ancestry from multiple source populations. We discuss how recent research on genetic admixture has extended our understanding of the distribution of human genetic variation.

**Figure 1.**
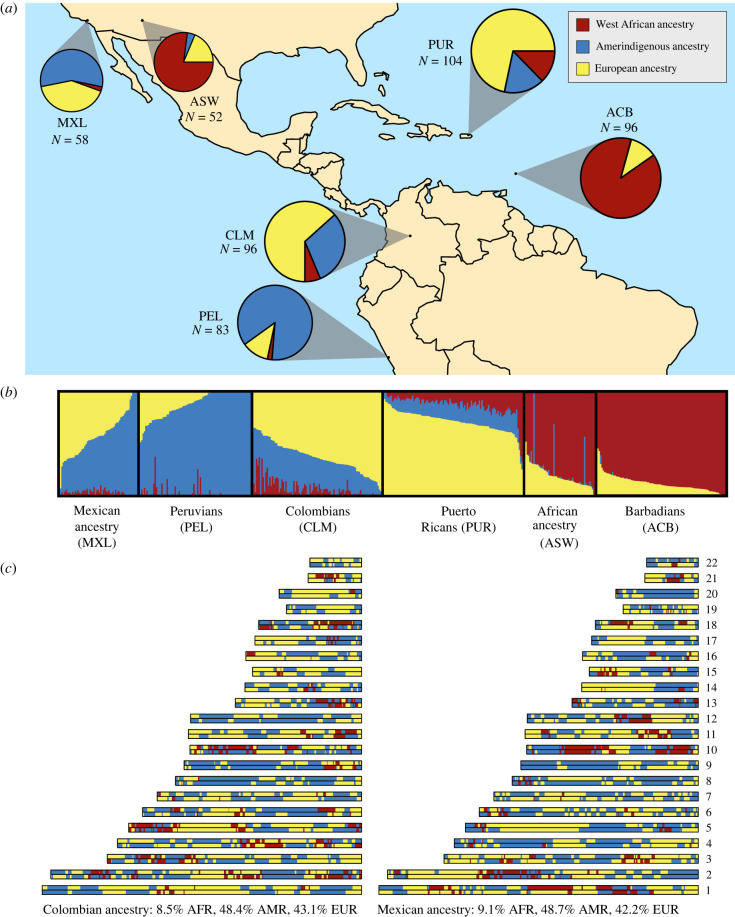
Ancestry in admixed populations varies at multiple genetic scales, with variance among individuals and within individual genomes. We show examples of global and local ancestry inferred from phased 1000 Genomes Project data for populations of the Americas and Caribbean. Global ancestry was estimated using unsupervised ADMIXTURE analysis, including additional populations of European (Iberians (IBS) and Tuscans in Italy (TSI)) and West African (Esan (ESN), Mandinka (GWD), Mende (MSL) and Yoruba (YRI)) ancestry for reference. We show (*a*) population-level and (*b*) individual-level estimates of global ancestry across Mexican ancestry (MXL), Peruvian (PEL), Colombian (CLM), Puerto Rican (PUR), African ancestry (ASW) and Barbadian (ACB) populations; barplots illustrating these estimates for *K* = 3 were made using pong [6]. (*c*) Local ancestry as inferred by RFMix [7] for two example individuals (HG01149 and NA19776) who have similar global ancestry proportions, and belong to the CLM and MXL populations, respectively. For these analyses, we retained only SNPs marked ‘PASS’ and removed all individuals who were noted to have an up to third degree relative in the 1000 Genomes Project phase 3 pedigree file, leaving 998 individuals for analysis. We then filtered SNPs for missingness (greater than 5%) and low minor allele frequency (less than 1%) across all populations, and Hardy–Weinberg disequilibrium (*p*-value < 0.000001) within populations. For our ADMIXTURE analyses, we also removed SNPs in linkage disequilibrium (using the PLINK command – indep-pairwise 50 10 0.1), which left 698 408 SNPs for analysis. We ran the ADMIXTURE algorithm for *K* = 3 unsupervised using the default settings and a random seed. Pong identified a single mode across 30 replicates. To estimate local ancestry, we used the missingness, minor allele frequency and Hardy–Weinberg filtered phased genotype dataset. We designated individuals with high levels (over 99%) of global West African (AFR), Amerindigenous (AMR) and European (EUR) ancestry, as determined by our ADMIXTURE analysis, as reference groups for those respective ancestries. We ran RFMix v. 2.03 for the target Colombian and Mexican ancestry individuals using the HapMap GRCh37 genetic map lifted over to GRCh38, a maximum of two expectation-maximization iterations, and otherwise default parameters. (Online version in colour.)

Beyond the allele-frequency-based summaries of variation studied by Lewontin [1], variation in admixed populations can be summarized based on ancestry from source populations. These ancestry patterns may vary between admixed populations formed by the same source populations, between individuals within an admixed population, and across loci within an admixed individual (figure 1). Population geneticists have long recognized that studying admixed human groups provides opportunities to learn about evolutionary forces [2,3]. Despite this early interest, inclusion of admixed populations in genetic studies is variable by research goal. Whereas the demographic and selective histories of admixed populations are well-studied, phenotypic and medical studies of admixed populations have lagged behind relative to studies of single-ancestry populations. For example, admixed populations are underrepresented in biobank datasets [7,8]. The lack of medical genomic samples and the frequent need for admixture-specific methods lead to admixed populations often being excluded from these studies [7–10]. Additionally, in practice, defining admixture in humans is highly context dependent, affected by social structures that influence population or self identification, as well as methodological limits on detecting admixture from genomic data (box 1).

Box 1. **Defining admixed populations.**

Discussions of genetic admixture are, implicitly or explicitly, predicated on the idea that meaningful genetic differences exist between discretized human groups. In practice, the term ‘admixed’ can vary to encompass a range of spatial and temporal processes of gene flow between previously isolated groups. In this review, as noted in §1, we have focused on recent admixture occurring within the past tens of generations. However, extensive gene flow between groups is a hallmark of recent human evolution; when examined in enough detail, nearly all populations can be described as descended from a combination of multiple ancestries. Similar to other discussions on delineating population definitions and boundaries, because there are no strict criteria that determine which populations should be considered admixed from a genetic perspective, classification of a population as admixed is often dependent on the context of the line of inquiry [11–14]. Additionally, while the effects of genetic admixture can be observed in individual genomes, it is conceptualized as a demographic process that acts on populations. For example, for a recent two-way admixture pulse between populations A and B, high variance in these ancestry components across individuals is expected; under neutrality, there will be individuals in the admixed population that derive 0%, and those that derive 100%, of their ancestry from population A (figure 1) [15,16]. As demonstrated in the bottom panel of figure 1, any individual locus in a genome from an admixed population can only contribute partially to inference of the demographic history of the admixed group due to the substantial variance in how ancestral diversity is distributed across genomes from an admixed population.

Methodological issues and patterns of genetic diversity are not the only factors shaping our understanding of genetic admixture; a long record of societies’ and scientists’ use of largely superficial characteristics to classify human groups also plays a role. Geneticists and anthropologists have long wrestled with the various field-specific and lay definitions of ‘population’, ‘ancestry’, ‘ethnicity’ and ‘race’, which interact and intersect with each other in complicated ways [11–14,17]. In discussions of human genetic admixture, it becomes especially important to emphasize that these categories do not map onto each other one-to-one, and that race and ethnicity, in particular, describe classifications based on social phenomena. The variation in ancestry within individuals in admixed populations, shown in figure 1, illustrates this and can be an effective tool in illustrating the difference between genetic ancestry, phenotypes and self-identified race and/or ethnicity.

Many population genetics methods and analytical results are based on assumptions about populations that do not hold under recent admixture. Under a model of isolation, metrics of genomic diversity often have well-defined theoretical expectations with respect to fundamental parameters of the population's evolutionary history. However, many of these relationships are unclear, with admixture introducing blocks of linked ancestral haplotypes each with potentially different patterns of variation based on the history of their source populations. That is, admixture changes both linkage structure and allele frequency distributions, which is often not accounted for in traditional inference methods developed without consideration of admixture.

Studying the ancestry patterns of present-day admixed groups has revealed information about the demographic histories of their source populations, including those that are uncommon in unadmixed form today [18,19]. For example, high-resolution genetic maps have been constructed based on the frequency of estimated local ancestry switchpoints (i.e. where local ancestry changes from one source to another along a single chromosome), which contains information about recombination rates along the genome [20,21]. Admixed genomes have also enabled the discovery of variant–trait associations and improvements in genetic risk prediction models beyond the associations identified and predictions that have been made using the ancestral populations [22–26]. Recent methodological improvements have increased the efficiency and performance of local ancestry calling (i.e. the assignment of genomic segments to their population of origin; some early scalable algorithmic implementations are given in [7,27–29]; figure 1*c*). These advances have enabled the use of local ancestry patterns in admixed populations to infer demographic history, adaptation and the genetic bases of complex traits.

Here, we consider three inferential problems based on studying patterns of genetic variation produced by admixture: inference of population history, identifying adaptive mutations and complex trait associations and prediction using admixed genomes. We summarize recent progress in the field, highlight as yet unresolved issues, and outline potential avenues of future research on the genetics of admixed populations. We focus on recent admixture between modern human populations, roughly corresponding to admixed populations founded within the last tens of generations; Witt *et al*. in this issue consider ancient admixture events with archaic humans and their consequences for human genetic variation [31].

## 2. Estimating genetic diversity and ancestry in admixed populations

Well before polymorphism data could be generated at a genome-wide scale, several methods of measuring genetic diversity had already been proposed, including heterozygosity and nucleotide diversity [32,33]. By connecting these to theoretical population genetics models, summaries of genetic variation can provide insight into the evolutionary forces acting on populations. However, inferring population history from genetic data is highly dependent on how groups are defined, a choice made by the researchers (box 1). Recent admixture complicates the quantification and analysis of genetic diversity, and can, therefore, affect traditional summaries of diversity in unexpected ways.

In his 1972 paper, Lewontin discusses his choices of a genetic diversity measure at some length, ultimately settling on one that is analogous to heterozygosity [1]. Relevant to genetic admixture, Lewontin specifically notes that:

‘a collection of individuals made by pooling two populations ought always to be more diverse than the average of their separate diversities, unless the two populations are identical in composition' (p. 338).

In this statement, Lewontin describes expectations of diversity in a set of pooled haplotypes originating from individuals of distinct ancestries, as might result from sampling schemes that combine populations in genetic analysis. This quote also gives insight into how admixture may impact the measures of genetic variation that Lewontin considers. These ideas were revisited in a recent study that explores patterns of heterozygosity in admixed populations [34]. The authors theoretically demonstrate that the heterozygosity of an admixed population is predicted by the heterozygosities of its source populations, the *F*_ST_ between them and the admixture contributions [34]. *F*_ST_, which has taken the place of entropy partitioning statistics that Lewontin [1] used, can also be informative about the parameters of the admixture process, as Boca and Rosenberg demonstrated [35]. These studies illustrate how traditional measures of genetic diversity can be repurposed to improve our understanding of the admixture process.

Beyond within- and between-population estimates of genetic diversity and ancestry, admixed populations introduce another class of summaries of genetic variation: tracts of the genome *within individuals* that originate from each ancestry source [15,17,36]. In figure 1, we illustrate three hierarchical categories of genetic ancestry variation in admixed populations from the 1000 Genomes Project [37] from the Americas, who have African, European and Amerindigenous ancestry. First, given similar continental source ancestries, admixed populations can vary in their average proportions from each source (figure 1*a*). Second, individuals within an admixed population may vary in their genome-wide, or ‘global’, ancestry proportions (figure 1*b*). Third, individuals with similar source ancestry contributions and admixture histories may vary by ‘local’ ancestry across genomic loci (figure 1*c*). At each level, these patterns of diversity contain information about admixture and post-admixture processes.

In practice, genetic ancestry of individuals from admixed populations is not fully known and is inferred, often using reference panels that are collated to represent the source populations [4,27–30,38]. In the following sections, we discuss aspects of human evolution that are commonly inferred from patterns of genetic variation in admixed populations, particularly genetic ancestry. The performance of these methods is predicated on accurate estimates of global and local ancestry.

The quality of ancestry estimates depends on a variety of sampling and evolutionary scenarios [39]. A recent study of the admixed Ashkenazi Jewish population noted that the lack of differentiation between European and Middle Eastern haplotypes made accurate local ancestry inference challenging, reducing their power to infer the parameters of the admixture process [40] and demonstrating the complexity in defining admixed populations, as these populations are often not considered admixed. The authors suggest that these issues might be mitigated by incorporating uncertainty in local ancestry estimates into complex demographic scenarios. Lawson *et al*. [39] demonstrate multiple avenues for potential over- or misinterpretation of global ancestry estimates from a commonly used suite of model-based methods based on the Pritchard–Stephens–Donnelly model of mixed membership across latent clusters. For example, they found that multiple qualitatively different evolutionary scenarios produced similar global ancestry estimates in the admixed population, and uneven sample sizes between populations may influence ancestry estimates. Notably, many methods, especially for local ancestry, rely on the use of reference panels of modern populations as proxies for the source populations, which may not fully represent the populations that existed at the time of admixture, and have uneven global representation.

## 3. Inferring population history

The admixture history of a population, such as the timing and source contribution levels, leaves predictable patterns of genetic variation within and between individuals from the admixed population [15,16,37,41–43]. Empirical genetic analyses can, therefore, be used to infer the histories that produced observed genetic variation.

Under a simple admixture scenario, the allele frequency of a locus in the admixed population is expected to be the average of the allele frequencies in source populations weighted by their contribution levels [44–46]. That is, the admixture contribution levels from the sources can be estimated from the allele frequencies of the admixed and source populations. Estimation of ancestry proportions under this model of admixture often relies on identifying a subset of loci with particularly large allele frequency differences between the source populations, known as Ancestry Informative Markers (AIMs) [47]. With further developments in genome sequencing increasing the density of loci across genomes, recent methods often incorporate linkage information or model small allele frequency changes over many loci, producing estimates of global ancestry proportions, as well as local ancestry along an admixed individual's genome [4,27–30,38]. Mechanistic models of admixture complement empirical studies to improve our intuition of admixture dynamics and interpretation of empirical results [15,42,48–51]. Related model-based inference frameworks have been developed to estimate parameters of population history.

Patterns of global and local ancestry within and between individuals are informative about admixture histories. For example, over time, recombination tends to break up local ancestry tracts; therefore, longer tracts generally indicate more recent contributions from source populations to an admixed population and may be used to infer the timing of admixture [36,42,52–58]. Similarly, as random mating leads to the averaging of ancestry proportions across individuals as they produce the next generation, the variance in global ancestry within the admixed population decreases over time as well [15]. Summaries of variation that are not explicitly based on local or global ancestry, such as linkage disequilibrium, can also be informative of the timing of admixture as populations with differentiated allele frequencies mix. With two-way admixture, high-frequency variants from each source will be strongly correlated with each other in the first-generation admixed population, regardless of their respective locations in the genome and degree of physical linkage. Over time, recombination will erode these correlations to generate a pattern of non-random association of pairs of loci that decay over genomic distance. Several methods leverage these characteristic decay curves to estimate the age of a pulse of admixture [4,59,60], and extensions of these methods infer admixture parameters under models that include continuous gene flow, multiple waves or assortative mating [61–63].

Similarly, sociocultural practices that govern mate choice or sex-specific contributions from the source populations will leave signatures in patterns of genetic ancestry. Individual behaviours such as mating preferences or long-range migration can exhibit ancestry biases in which the ancestry patterns in the subset of the population that migrates are not representative of the whole admixed population, potentially driven by correlations between ancestry and visible traits like skin pigmentation or socioeconomic differences [64–69]. Simple models of admixture often assume that individuals mate randomly; however, admixed human populations show evidence of positive assortative mating, with mating pairs often correlated in global ancestry proportion [67,70–72]. Recent methods have sought to test for ancestry-based assortative mating by developing frameworks to infer parental ancestries from phased haplotypes within a single individual [73–75]. When not accounted for, nonrandom mating patterns can bias inference of admixture parameters [62,76].

Additionally, based on the sex-specific inheritance of the X chromosome (where females inherit two copies, one from each parent, while males inherit one X chromosome maternally and their Y chromosome paternally), comparisons of X-chromosomal and autosomal ancestry proportions have been used to infer sex-biased admixture in ancient and modern human populations [49,50,77–80]. These differences in female and male contribution levels from the sources may be indicative of complex social interactions that govern mating behaviors between the admixing human populations, such as dominance structures associated with colonization.

Differences in ancestry proportion across the geographical span of a population or populations with shared ancestry components have been used to infer ancestry-biased migration patterns, which may be driven by social cues. For example, ancestry-biased migration, often combined with other mating dynamics, has been proposed as a process shaping regional variation in African ancestry proportions across the USA [65,81,82]. Similarly, temporal changes in ancestry proportion within a population may be caused by time-varying social dynamics. Spear *et al*. [69] found a significant increase in Amerindigenous ancestry in Mexican American populations over time, potentially owing to differences in ancestry in the migrating population over time and fecundity correlated with ancestry.

Sufficiently accounting for these spatial and temporal dynamics of the admixture process presents an exciting challenge. One solution to address admixture processes that vary over space or time involves simulation-based demographic inference frameworks, such as approximate Bayesian computation and machine learning-based approaches. For example, MetHis is an approximate Bayesian computation-based approach for inference under complex two-way admixture models [48,83]. An advantage of simulation-based demographic inference methods over models that use a likelihood is that they can handle arbitrarily complicated admixture scenarios, accommodate any calculable feature of genomic data (such as tracts that are identical by descent (IBD) and runs of homozygosity (ROH)), and even conduct summary-statistic-free inference [84]. Continued work to extend these methods will enable disentangling the myriad of historical, evolutionary and socio-cultural factors contributing to human admixture processes.

Studying the genomes of admixed populations can also provide insight into the genetic origins and demographic histories of their founding populations, particularly for source ancestries that are no longer commonly represented by an extant single-ancestry population [18,19]. An increasingly popular approach is to first estimate local ancestry, then separately apply classic single-population methods on the subsets of the genome that are inferred to be from each source. This is exemplified by the ancestry-specific PCA (ASPCA) method, which performs PCA separately for each contributing source ancestry, as identified by local ancestry inference methods. This approach has revealed previously unappreciated variation in the European and Amerindigenous ancestry sources of admixed Latinos across Mexico [85], the Caribbean [86] and South America [87].

Local ancestry inference can also be used to unravel source-specific historical population size dynamics. The process of admixture often involves bottlenecks at the time of founding, the timing and strength of which Browning *et al*. [88] demonstrated can be inferred using ancestry-specific IBD. This approach combines estimates of local ancestry and IBD for admixed groups to estimate the past effective population sizes of each of the source ancestries. They found ancestry-specific population size changes, including  variable bottleneck severity .

Moving forward, combining ancestry-based inference with patterns of homozygosity and IBD may help elucidate these complex and dynamic population histories. For example, homozygosity and IBD are shaped by the relationships between mating pairs, which are in turn influenced by sociocultural processes [65,67,86,89,90]. However, we lack theoretical expectations for the distributions of ROH and IBD segments after admixture, which may break up local patterns of homozygosity while also involving major changes in genome-wide variation due to the mixing of previously isolated populations. Recent empirical explorations suggest that, in particular, ROH in admixed populations reflect both contributions from source populations and post-admixture population dynamics.

## 4. Detecting selection

Adaptation to biotic and abiotic environments leaves signatures in patterns of human genetic variation that can be used to identify adaptive loci and infer their selection history [91–94]. However, admixture can confound this inferential process and obscure the detection of genomic targets of selection by producing genetic signatures that are classically interpreted as signatures of selection [95–98]. Additionally, the long-range geographical movement of people associated with recent admixture may introduce novel selective pressures. Under certain scenarios, selection may indeed be easier to detect in admixed populations than in single-ancestry populations with the additional information provided by ancestry patterns [99–102]. That is, inferring selection from admixed genomes poses unique challenges, but also opportunities for new insights into human adaptation.

As described previously, admixed populations are often considered as a linear combination of their sources such that the expected allele frequency of a locus is an average of the allele frequencies in each source population at that locus weighted by their proportional contribution to the admixed population. Loci that dramatically differ from this expectation are candidates for loci under selection (reviewed in Adams & Ward [45], and Chakraborty [3]).

Outlier methods have been used to detect selection with a variety of summary statistics in single-ancestry populations, including early work by Lewontin and Krakauer [103], and more recently, IBD or ROH. However, non-equilibrium demographic processes such as bottlenecks and gene flow can change the distribution of these statistics across the genome, leading to false positives or complicating interpretation of these outlier methods [104–107]. When using methods not specifically developed for admixed populations, admixture can lead to both increased false-positive rates and decreased power to detect both pre-admixture selection (i.e. selection that happened in the source populations) and post-admixture selection [96].

Recent methods often leverage ancestry information to detect post-admixture adaptation, independently based on ancestry distributions, or in combination with other classic summary statistics [99–102,108–110]. When selective pressures are shared between admixed populations and one of their sources, admixture-mediated adaptation may occur through contributions of an adaptive allele from that source population. This may be a particularly rapid mode of adaptation because the allele is often introduced into the admixed population at intermediate to high frequency (proportional to the admixture contribution from that source), decreasing stochastic loss. If the adaptive allele is common in one source population but rare in the other(s), then as that allele rises in frequency in the admixed population, so will the corresponding local ancestry at that locus. This observation has led to a common method to detect post-admixture selection: scanning for outliers in local ancestry compared to genome-wide ancestry.

**Figure 2.**
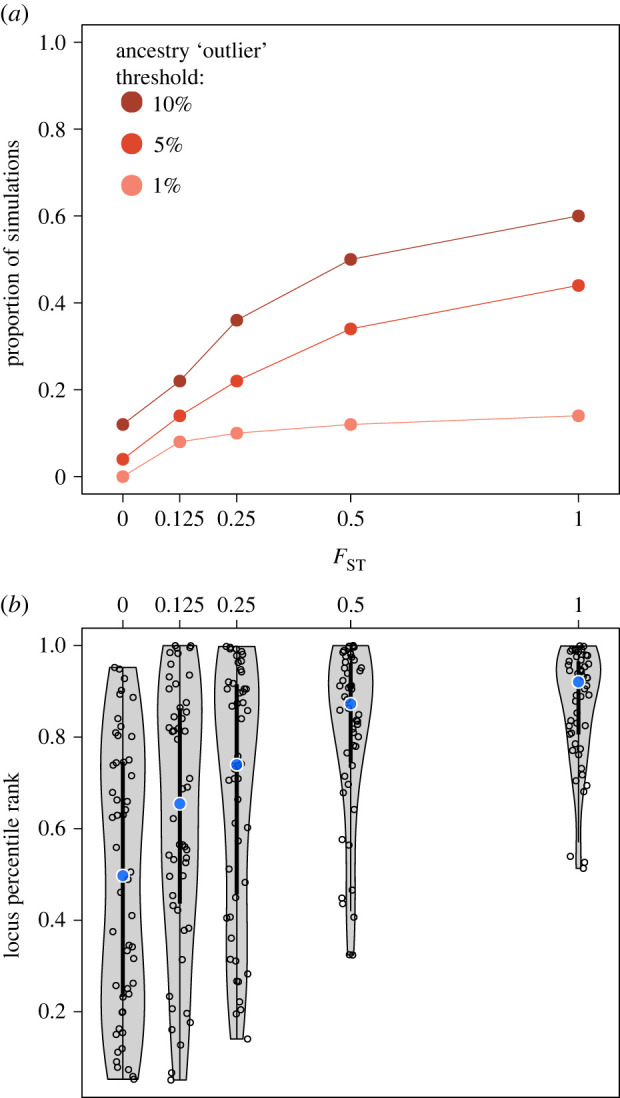
Ancestry outlier tests for post-admixture selection are underpowered when source differentiation is low. We examine how *F*_ST_ between two source populations at a selected locus affects the power of a local ancestry outlier approach to detect selection. Whole-genome simulations were conducted in SLiM [114]. We simulated 50 sets of 10 000 individuals under a two-way admixture model with equal contributions from the sources, with Population A contributing an allele that is under strong selection (*s* = 0.05) in the admixed population for 12 generations. For increasing values of *F*_ST_ along the *x* axis, we plot (*a*) the proportion of simulations in which the selected locus would be classified as an ‘outlier’ in local ancestry frequency from Population A for multiple genome-wide thresholds, and (*b*) the rank of the selected locus among all loci genome-wide for ancestry from Population A. Even with relatively strong selection and complete differentiation between source ancestries (i.e. *F*_ST_ = 1) at the selected locus, it frequently failed to appear as a Population A ancestry outlier, potentially because selection had not had long enough to act, resulting in other loci having higher local ancestry frequencies in the population by chance. Similarly, the rank (with all loci ordered by frequency of local ancestry from Population A) of the selected locus increases with increasing differentiation between source populations at the locus. We simulated 6 diploid individuals per source population, and use the (potentially multiple) allele frequency combinations that produce the five values of *F*_ST_ plotted, specifying that Population A's frequency was equal to or higher than Population B's. From these, we randomly chose a starting allele frequency combination for the source populations for each of the 50 simulations. (Online version in colour.)

Empirical studies have identified numerous candidate regions under selection post-admixture using ancestry outlier methods [108,110–112]; however, this approach has several limitations. The distribution of local ancestry within a population is influenced by a complex interplay of selective and demographic histories, and current theoretical understanding is limited, making the choice of cutoff for identifying outliers somewhat arbitrary [113]. More fundamentally, an ancestry-outlier approach is only suitable in situations where the allele frequencies in the source populations differ substantially, which couples allele frequency changes with a single source's ancestry. In figure 2 we demonstrate this coupling by simulating admixture with equal contributions from two sources, followed by 12 generations of strong selection (*s* = 0.05) at the adaptive locus; the proportion of simulations in which the adaptive locus is an outlier increases with increasing *F*_ST_ between the sources. Additionally, the power of outlier approaches to localize adaptive loci depends on the length of the admixture tract containing the locus, and therefore the selection history. Finally, while useful for identifying adaptive loci, these methods must be combined with other information or simulations to infer parameters of the population's history such as the strength or timing of selection.

Ongoing work extends initial implementations of ancestry-outlier approaches to study post-admixture selection, and often uses simulations to improve interpretation and test power [99–102,115,116]. These methods have recovered classic examples of selected loci from the genomes of admixed populations and inferred the timing, strength and repeatability of selection under different scenarios. For example, our work in Hamid *et al*. [100] found signatures of adaptation to malaria via the *DARC* gene in the admixed population of Cabo Verde based on long, high frequency African ancestry tracts. Hamid *et al*. [100] further used simulation-based inference to infer the strength of selection. This study's findings reinforced others that have identified post-admixture selection pressure to retain African ancestry at *DARC*, a known malaria susceptibility locus, in multiple admixed populations on multiple timescales [99,112,117–119]. It also provides an example of combining ancestry-specific summary statistics with simulations to both localize selection and infer parameters of the selection history.

While these recent studies using empirically driven summary statistics have proven informative in certain scenarios, more work is needed to develop expectations of the distributions of ancestry under models of selection with admixture. Indeed, recent work has suggested perhaps unexpected relationships between ancestry tract lengths, allele frequencies and selection history, emphasizing the need for additional theory [120].

## 5. Understanding complex trait architecture and predicting genetic traits

For decades, human genetics research has aspired to make personalized medical therapies a reality by improving the prediction of traits from genetic data; while progress has been made on the genetic prediction of traits in recent years, its potential for making personalized medicine a reality may only be currently applicable to individuals of European ancestry [121]. Genome-wide association studies (GWAS) have been the standard framework for studying the genetic basis of complex traits for over 15 years, in which variants across the genome are tested individually for statistical association with a trait of interest. GWAS studies have also formed the statistical foundation for polygenic scores (PGS), in which complex quantitative traits (e.g. height or cholesterol level) are predicted under Fisher's infinitesimal model using the sum of an individual's observed genotypes weighted by GWAS-inferred effect sizes.

Admixture complicates the identification of genetic underpinnings of complex traits. For example, GWAS generally assume that there are no systematic differences in the genetic variation of the study cohort except in those variants that underlie the trait of interest. Yet patterns of ancestry vary widely across individuals within an admixed population, both at the genome-wide level and within regions of the genome, as shown in figure 1. Local ancestry block structures induced by admixture processes cannot be controlled for using genome-wide ancestry (e.g. principal components) as covariates, as is standard practice in GWAS, and as a result GWAS of admixed populations may have inflated error rates [10,22,122,123]. That is, admixture introduces complex population structure and linkage blocks that, if unaccounted for, can identify false-positive variant–trait associations. Recent research has shown that variant-level effect sizes on a given trait estimated from GWAS tend to be ancestry- or even study-specific [124–127]. This severely limits the ability to use effect sizes estimated in a sample from one ancestry to predict trait levels in a sample from a different ancestry, which generally results in poor trait prediction accuracy for individuals who were not part of the discovery GWAS, even if from the same ancestry [69,124,126,128].

Increasingly, research suggests that by excluding individuals from admixed populations (as well as from non-admixed minority populations), geneticists are discarding a rich source of genomic information [26]. PGS accuracy could be improved with more comprehensive sequencing of cohorts of non-European ancestry [7,125,129,130], but must be coupled with new methods tailored to admixed populations and the patterns of linkage disequilibrium patterns and allele frequency variation that arise from their population histories (see also Fish *et al*. [131]). Furthermore, source ancestry contributions to admixed populations and their dynamics within admixed populations can change over time, leading to temporal variation in effect size estimates [69]. All of these factors can contribute to a loss of predictive power in individuals of admixed ancestry, even when accounting for local ancestry and using high-quality effect size estimates for all source ancestries [132,133].

In an effort to address these challenges for predicting traits in admixed populations, new frameworks are being developed to improve the performance of PGS in individuals from admixed populations, such as including local ancestry-based principal components to correct for heterogeneous patterns of population structure along the genome or subdividing the cohort by genome-wide ancestry and taking a meta-analysis approach [121].

## 6. Conclusion

Though it was not the focus of his paper, Lewontin [1] acknowledged the role of admixture in shaping distributions of genetic variation and included admixed populations in his analyses (see also box 1). In the intervening fifty years, population genetics research has continued to shed light on the importance of admixture processes for genetic variation and complex trait architectures. In certain scenarios, studying admixed populations may provide insight into general human evolutionary processes (for example, recombination as in [20,21]) and history beyond admixture itself because of the added information from ancestry-based statistics.

Multiple future directions in research on admixture will extend our understanding of human evolution and the distribution of human genetic variation. First, there is a need for more theory regarding how natural selection interacts with admixed population histories (but see [120]). Figure 2, as well as multiple recent studies [101,102], show that common summary statistics to detect selection in admixed populations have variable power and often unclear interpretations. Moving beyond simple implementations of ancestry-outlier approaches, which provide a list of candidate loci, may also be useful for developing methods to infer the selective history of adaptive loci. Second, the study of admixed populations is often based on contrasting genetic variation in admixed populations against that of reference populations for source ancestries, even if accurate references are not available. Reference-free methods have proven useful for estimating global ancestry, for example in unsupervised implementations of ADMIXTURE and STRUCTURE, yet remain rare for local ancestry assignment (but see [134]). Finally, methods have thus far focused on positive selection primarily at single loci, and more work is needed to study other directions or genetic architectures under selection, such as background and polygenic selection. An important step for interpreting signals of adaptation is understanding the genetic basis of traits. Towards this goal, multiple recent studies have focused on methods for predicting quantitative traits in admixed populations [69,125,128,129,135], and offer new insight into how admixture linkage disequilibrium specifically confounds the identification of shared genetic associations. Prioritization of sampling from admixed populations for association studies would increase power to accurately estimate effect sizes for these groups rather than relying on GWAS results from proxies for their ancestral sources [121,122,129].

## Data Availability

This article has no additional data.

## References

[1] Lewontin RC. 1972 The Apportionment of Human Diversity. In Evolutionary biology: vol. 6 (eds T Dobzhansky, MK Hecht, WC Steere), pp. 381-398. New York, NY: Springer US.

[2] Cavalli-Sforza LL, Menozzi P, Piazza A. 1994 The history and geography of human genes. Princeton, NJ: Princeton University Press.

[3] Chakraborty R. 1986 Gene admixture in human populations: models and predictions. Am. J. Phys. Anthropol. **29**, 1-43. (10.1002/ajpa.1330290502)

[4] Hellenthal G, Busby GBJ, Band G, Wilson JF, Capelli C, Falush D, Myers S. 2014 A genetic atlas of human admixture history. Science **343**, 747-751. (10.1126/science.1243518) 24531965 PMC4209567

[5] Korunes KL, Goldberg A. 2021 Human genetic admixture. PLoS Genet. **17**, e1009374. (10.1371/journal.pgen.1009374) 33705374 PMC7951803

[6] Behr AA, Liu KZ, Liu-Fang G, Nakka P, Ramachandran S. 2016 pong: fast analysis and visualization of latent clusters in population genetic data. Bioinformatics **32**, 2817-2823. (10.1093/bioinformatics/btw327) 27283948 PMC5018373

[7] Maples BK, Gravel S, Kenny EE, Bustamante CD. 2013 RFMix: a discriminative modeling approach for rapid and robust local-ancestry inference. Am. J. Hum. Genet. **93**, 278-288. (10.1016/j.ajhg.2013.06.020) 23910464 PMC3738819

[8] Popejoy AB, Fullerton SM. 2016 Genomics is failing on diversity. Nature **538**, 161-164. (10.1038/538161a) 27734877 PMC5089703

[9] Ben-Eghan C, Sun R, Hleap JS, Diaz-Papkovich A, Munter HM, Grant AV, Dupras C, Gravel S. 2020 Don't ignore genetic data from minority populations. Nature **585**, 184-186. (10.1038/d41586-020-02547-3) 32901124

[10] Knowler WC, Williams RC, Pettitt DJ, Steinberg AG. 1988 Gm3;5,13,14 and type 2 diabetes mellitus: an association in American Indians with genetic admixture. Am. J. Hum. Genet. **43**, 520-526. 3177389 PMC1715499

[11] Medina-Gomez C et al. 2015 Challenges in conducting genome-wide association studies in highly admixed multi-ethnic populations: the Generation R Study. Eur. J. Epidemiol. **30**, 317-330. (10.1007/s10654-015-9998-4) 25762173 PMC4385148

[12] Rosenberg NA, Mahajan S, Ramachandran S, Zhao C, Pritchard JK, Feldman MW. 2005 Clines, clusters, and the effect of study design on the inference of human population structure. PLoS Genet. **1**, e70. (10.1371/journal.pgen.0010070) 16355252 PMC1310579

[13] Fujimura JH, Rajagopalan R. 2011 Different differences: the use of ‘genetic ancestry’ versus race in biomedical human genetic research. Soc. Stud. Sci. **41**, 5-30. (10.1177/0306312710379170) 21553638 PMC3124377

[14] Ali-Khan SE, Krakowski T, Tahir R, Daar AS. 2011 The use of race, ethnicity and ancestry in human genetic research. Hugo J. **5**, 47-63. (10.1007/s11568-011-9154-5) 22276086 PMC3237839

[15] Van Arsdale AP. 2019 Population demography, ancestry, and the biological concept of race. Annu. Rev. Anthropol. **48**, 227-241. (10.1146/annurev-anthro-102218-011154)

[16] Verdu P, Rosenberg NA. 2011 A general mechanistic model for admixture histories of hybrid populations. Genetics **189**, 1413-1426. (10.1534/genetics.111.132787) 21968194 PMC3241432

[17] Long JC. 1991 The genetic structure of admixed populations. Genetics **127**, 417-428. (10.1093/genetics/127.2.417) 2004712 PMC1204369

[18] Mathieson I, Scally A. 2020 What is ancestry? PLoS Genet. **16**, e1008624. (10.1371/journal.pgen.1008624) 32150538 PMC7082057

[19] Schroeder H et al. 2018 Origins and genetic legacies of the Caribbean Taino. Proc. Natl Acad. Sci. USA **115**, 2341-2346. (10.1073/pnas.1716839115) 29463742 PMC5877975

[20] Ávila-Arcos MC et al. 2020 Population history and gene divergence in Native Mexicans inferred from 76 human exomes. Mol. Biol. Evol. **37**, 994-1006. (10.1093/molbev/msz282) 31848607 PMC7086176

[21] Wegmann D et al. 2011 Recombination rates in admixed individuals identified by ancestry-based inference. Nat. Genet. **43**, 847-853. (10.1038/ng.894) 21775992 PMC8582322

[22] Hinch AG et al. 2011 The landscape of recombination in African Americans. Nature **476**, 170-175. (10.1038/nature10336) 21775986 PMC3154982

[23] Atkinson EG et al. 2021 Tractor uses local ancestry to enable the inclusion of admixed individuals in GWAS and to boost power. Nat. Genet. **53**, 195-204. (10.1038/s41588-020-00766-y) 33462486 PMC7867648

[24] Pasaniuc B et al. 2011 Enhanced statistical tests for GWAS in admixed populations: assessment using African Americans from CARe and a Breast Cancer Consortium. PLoS Genet. **7**, e1001371. (10.1371/journal.pgen.1001371) 21541012 PMC3080860

[25] Seldin MF, Pasaniuc B, Price AL. 2011 New approaches to disease mapping in admixed populations. Nat. Rev. Genet. **12**, 523-528. (10.1038/nrg3002) 21709689 PMC3142784

[26] Zaitlen N et al. 2014 Leveraging population admixture to characterize the heritability of complex traits. Nat. Genet. **46**, 1356-1362. (10.1038/ng.3139) 25383972 PMC4244251

[27] Lin M, Park DS, Zaitlen NA, Henn BM, Gignoux CR. 2021 Admixed populations improve power for variant discovery and portability in genome-wide association studies. Front. Genet. **12**, 829. (10.3389/fgene.2021.673167) PMC8181458 34108994

[28] Price AL et al. 2009 Sensitive detection of chromosomal segments of distinct ancestry in admixed populations. PLoS Genet. **5**, e1000519. (10.1371/journal.pgen.1000519) 19543370 PMC2689842

[29] Baran Y et al. 2012 Fast and accurate inference of local ancestry in Latino populations. Bioinformatics **28**, 1359-1367. (10.1093/bioinformatics/bts144) 22495753 PMC3348558

[30] Brisbin A et al. 2012 PCAdmix: principal components-based assignment of ancestry along each chromosome in individuals with admixed ancestry from two or more populations. Hum. Biol. **84**, 343-364. (10.3378/027.084.0401) 23249312 PMC3740525

[31] Witt KE, Villanea F, Loughran E, Huerta-Sanchez E.. 2022 Apportioning archaic variants among modern populations. Phil. Trans. R. Soc. B **377**, 20200411. (10.1098/rstb.2020.0411.) PMC9014186 35430882

[32] Watterson GA. 1975 On the number of segregating sites in genetical models without recombination. Theor. Popul. Biol. **7**, 256-276. (10.1016/0040-5809(75)90020-9) 1145509

[33] Nei M, Li WH. 1979 Mathematical model for studying genetic variation in terms of restriction endonucleases. Proc. Natl Acad. Sci. USA **76**, 5269-5273. (10.1073/pnas.76.10.5269) 291943 PMC413122

[34] Boca SM, Huang L, Rosenberg NA. 2020 On the heterozygosity of an admixed population. J. Math. Biol. **81**, 1217-1250. (10.1007/s00285-020-01531-9) 33034736 PMC7710588

[35] Boca SM, Rosenberg NA. 2011 Mathematical properties of *F*_st_ between admixed populations and their parental source populations. Theor. Popul. Biol. **80**, 208-216. (10.1016/j.tpb.2011.05.003) 21640742 PMC3206961

[36] Gravel S. 2012 Population genetics models of local ancestry. Genetics **191**, 607-619. (10.1534/genetics.112.139808) 22491189 PMC3374321

[37] 1000 Genomes Project Consortium *et al*. 2015 A global reference for human genetic variation. Nature **526**, 68-74. (10.1038/nature15393) 26432245 PMC4750478

[38] Tang H, Coram M, Wang P, Zhu X, Risch N. 2006 Reconstructing genetic ancestry blocks in admixed individuals. Am. J. Hum. Genet. **79**, 1-12. (10.1086/504302) 16773560 PMC1474129

[39] Lawson DJ, van Dorp L, Falush D. 2018 A tutorial on how not to over-interpret STRUCTURE and ADMIXTURE bar plots. Nat. Commun. **9**, 1-11. (10.1038/s41467-018-05257-7) 30108219 PMC6092366

[40] Xue J, Lencz T, Darvasi A, Pe'er I, Carmi S. 2017 The time and place of European admixture in Ashkenazi Jewish history. PLoS Genet. **13**, e1006644. (10.1371/journal.pgen.1006644) 28376121 PMC5380316

[41] Pfaff CL et al. 2001 Population structure in admixed populations: effect of admixture dynamics on the pattern of linkage disequilibrium. Am. J. Hum. Genet. **68**, 198-207. (10.1086/316935) 11112661 PMC1234913

[42] Liang M, Nielsen R. 2014 The lengths of admixture tracts. Genetics **197**, 953-967. (10.1534/genetics.114.162362) 24770332 PMC4096373

[43] Liang M, Nielsen R. 2014 Understanding admixture fractions. bioRxiv, 008078. (10.1101/008078)

[44] Bernstein, F. 1931 Die geographische Verteilung der Blutgruppen und ihre anthropologische Bedeutung. In Comitato italiano per lo studio dei problemi della populazione. Rome, Italy: Instituto Poligrafico dello Stato.

[45] Adams J, Ward RH. 1973 Admixture studies and the detection of selection. Science **180**, 1137-1143. (10.1126/science.180.4091.1137) 4707061

[46] Long JC et al. 1991 Genetic variation in Arizona Mexican Americans: estimation and interpretation of admixture proportions. Am. J. Phys. Anthropol. **84**, 141-157. (10.1002/ajpa.1330840204) 2021190

[47] Shriver MD, Smith MW, Jin L, Marcini A, Akey JM, Deka R, Ferrell RE. 1997 Ethnic-affiliation estimation by use of population-specific DNA markers. Am. J. Hum. Genet. **60**, 957. 9106543 PMC1712479

[48] Buzbas EO, Verdu P. 2018 Inference on admixture fractions in a mechanistic model of recurrent admixture. Theor. Popul. Biol. **122**, 149-157. (10.1016/j.tpb.2018.03.006) 29604302 PMC6054545

[49] Goldberg A, Verdu P, Rosenberg NA. 2014 Autosomal admixture levels are informative about sex bias in admixed populations. Genetics **198**, 1209-1229. (10.1534/genetics.114.166793) 25194159 PMC4224161

[50] Goldberg A, Rosenberg NA. 2015 Beyond 2/3 and 1/3: the complex signatures of sex-biased admixture on the X chromosome. Genetics **201**, 263-279. (10.1534/genetics.115.178509) 26209245 PMC4566268

[51] Ewens WJ, Spielman RS. 1995 The transmission/disequilibrium test: history, subdivision, and admixture. Am. J. Hum. Genet. **57**, 455-464. (10.1002/ajmg.1320570319) 7668272 PMC1801556

[52] Pool JE, Nielsen R. 2009 Inference of historical changes in migration rate from the lengths of migrant tracts. Genetics **181**, 711-719. (10.1534/genetics.108.098095) 19087958 PMC2644959

[53] Pugach I, Matveyev R, Wollstein A, Kayser M, Stoneking M. 2011 Dating the age of admixture via wavelet transform analysis of genome-wide data. Genome Biol. **12**, R19. (10.1186/gb-2011-12-2-r19) 21352535 PMC3188801

[54] Jin W, Wang S, Wang H, Jin L, Xu S. 2012 Exploring population admixture dynamics via empirical and simulated genome-wide distribution of ancestral chromosomal segments. Am. J. Hum. Genet. **91**, 849-862. (10.1016/j.ajhg.2012.09.008) 23103229 PMC3487126

[55] Ni X, Yang X, Guo W, Yuan K, Zhou Y, Ma Z, Xu S. 2016 Length distribution of ancestral tracks under a general admixture model and its applications in population history inference. Sci. Rep. **6**, 20048. (10.1038/srep20048) 26818889 PMC4730239

[56] Ni X, Yuan K, Yang X, Feng Q, Guo W, Ma Z, Xu S. 2018 Inference of multiple-wave admixtures by length distribution of ancestral tracks. Heredity **121**, 52-63. (10.1038/s41437-017-0041-2) 29358727 PMC5997750

[57] Ni X, Yuan K, Liu C, Feng Q, Tian L, Ma Z, Xu S. 2019 MultiWaver 2.0: modeling discrete and continuous gene flow to reconstruct complex population admixtures. Eur. J. Hum. Genet. **27**, 133-139. (10.1038/s41431-018-0259-3) 30206356 PMC6303267

[58] Ioannidis AG et al. 2020 Native American gene flow into Polynesia predating Easter Island settlement. Nature **583**, 572-577. (10.1038/s41586-020-2487-2) 32641827 PMC8939867

[59] Moorjani P et al. 2011 The history of African gene flow into Southern Europeans, Levantines, and Jews. PLoS Genet. **7**, e1001373. (10.1371/journal.pgen.1001373) 21533020 PMC3080861

[60] Loh P-R, Lipson M, Patterson N, Moorjani P, Pickrell JK, Reich D, Berger B. 2013 Inferring admixture histories of human populations using linkage disequilibrium. Genetics **193**, 1233-1254. (10.1534/genetics.112.147330) 23410830 PMC3606100

[61] Pickrell JK, Patterson N, Loh P-R, Lipson M, Berger B, Stoneking M, Pakendorf B, Reich D. 2014 Ancient west Eurasian ancestry in southern and eastern Africa. Proc. Natl Acad. Sci. USA **111**, 2632-2637. (10.1073/pnas.1313787111) 24550290 PMC3932865

[62] Zaitlen N et al. 2017 The effects of migration and assortative mating on admixture linkage disequilibrium. Genetics **205**, 375-383. (10.1534/genetics.116.192138) 27879348 PMC5223515

[63] Zhou Y, Yuan K, Yu Y, Ni X, Xie P, Xing EP, Xu S. 2017 Inference of multiple-wave population admixture by modeling decay of linkage disequilibrium with polynomial functions. Heredity **118**, 503-510. (10.1038/hdy.2017.5) 28198814 PMC5564381

[64] Ruiz-Linares A et al. 2014 Admixture in Latin America: geographic structure, phenotypic diversity and self-perception of ancestry based on 7342 individuals. PLoS Genet. **10**, e1004572. (10.1371/journal.pgen.1004572) 25254375 PMC4177621

[65] Baharian S et al. 2016 The great migration and African-American genomic diversity. PLoS Genet. **12**, e1006059. (10.1371/journal.pgen.1006059) 27232753 PMC4883799

[66] Martin AR et al. 2017 An unexpectedly complex architecture for skin pigmentation in Africans. Cell **171**, 1340-1353.e14. (10.1016/j.cell.2017.11.015) 29195075 PMC5884124

[67] Korunes KL, Soares-Souza GB, Bobrek K, Tang H, Araújo II, Goldberg A, Beleza S. 2020 422766 Sex-biased admixture and assortative mating shape genetic variation and influence demographic inference in admixed Cabo Verdeans. bioRxiv. 422766. (10.1101/2020.12.14.422766) PMC9526050 35861404

[68] Kim J, Edge MD, Goldberg A, Rosenberg NA. 2021 Skin deep: the decoupling of genetic admixture levels from phenotypes that differed between source populations. Am. J. Phys. Anthropol. **175**, 406-421. (10.1002/ajpa.24261) 33772750 PMC8202736

[69] Spear ML, Diaz-Papkovich A, Ziv E, Yracheta JM, Gravel S, Torgerson DG, Hernandez RD. 2020 Recent shifts in the genomic ancestry of Mexican Americans may alter the genetic architecture of biomedical traits. Elife **9**, e56029. (10.7554/eLife.56029) 33372659 PMC7771964

[70] Risch N et al. 2009 Ancestry-related assortative mating in Latino populations. Genome Biol. **10**, R132. (10.1186/gb-2009-10-11-r132) 19930545 PMC3091325

[71] Sebro R, Hoffman TJ, Lange C, Rogus JJ, Risch NJ. 2010 Testing for non-random mating: evidence for ancestry-related assortative mating in the Framingham heart study. Genet. Epidemiol. **34**, 674-679. (10.1002/gepi.20528) 20842694 PMC3775670

[72] Zou JY, Park DS, Burchard EG, Torgerson DG, Pino-Yanes M, Song YS, Sankararaman S, Halperin E, Zaitlen N. 2015 Genetic and socioeconomic study of mate choice in Latinos reveals novel assortment patterns. Proc. Natl Acad. Sci. USA **112**, 13 621-13 626. (10.1073/pnas.1501741112) PMC4640764 26483472

[73] Zou JY, Halperin E, Burchard E, Sankararaman S. 2015 Inferring parental genomic ancestries using pooled semi-Markov processes. Bioinformatics **31**, i190-i196. (10.1093/bioinformatics/btv239) 26072482 PMC4765873

[74] Pei J, Zhang Y, Nielsen R, Wu Y. 2020 Inferring the ancestry of parents and grandparents from genetic data. PLoS Comput. Biol. **16**, e1008065. (10.1371/journal.pcbi.1008065) 32797037 PMC7449501

[75] Avadhanam S, Williams AL. 2022 Simultaneous inference of parental admixture proportions and admixture times from unphased local ancestry calls. bioRxiv, 475139. (10.1101/2022.01.05.475139)

[76] Goldberg A, Rastogi A, Rosenberg NA. 2020 Assortative mating by population of origin in a mechanistic model of admixture. Theor. Popul. Biol. **134**, 129-146. (10.1016/j.tpb.2020.02.004) 32275920 PMC7387155

[77] Bryc K et al. 2010 Genome-wide patterns of population structure and admixture in West Africans and African Americans. Proc. Natl Acad. Sci. USA **107**, 786-791. (10.1073/pnas.0909559107) 20080753 PMC2818934

[78] Goldberg A, Günther T, Rosenberg NA, Jakobsson M. 2017 Ancient X chromosomes reveal contrasting sex bias in Neolithic and Bronze Age Eurasian migrations. Proc. Natl Acad. Sci. USA **114**, 2657-2662. (10.1073/pnas.1616392114) 28223527 PMC5347611

[79] Ongaro L et al. 2019 The genomic impact of European colonization of the Americas. Curr. Biol. **29**, 3974-3986. (10.1016/j.cub.2019.09.076) 31735679

[80] Micheletti SJ et al. 2020 Genetic consequences of the transatlantic slave trade in the Americas. Am. J. Hum. Genet. **107**, 265-277. (10.1016/j.ajhg.2020.06.012) 32707084 PMC7413858

[81] Workman PL, Blumberg BS, Cooper AJ. 1963 Selection, gene migration and polymorphic stability in a U. S. White and Negro population. Am. J. Hum. Genet. **15**, 429-437. 14097237 PMC1932566

[82] Bryc K, Durand EY, Macpherson JM, Reich D, Mountain JL. 2015 The genetic ancestry of African Americans, Latinos, and European Americans across the United States. Am. J. Hum. Genet. **96**, 37-53. (10.1016/j.ajhg.2014.11.010) 25529636 PMC4289685

[83] Fortes-Lima CA, Laurent R, Thouzeau V, Toupance B, Verdu P. 2021 Complex genetic admixture histories reconstructed with Approximate Bayesian Computation. Mol. Ecol. Resour. **21**, 1098-1117. (10.1111/1755-0998.13325) 33452723 PMC8247995

[84] Flagel L, Brandvain Y, Schrider DR. 2019 The unreasonable effectiveness of convolutional neural networks in population genetic inference. Mol. Biol. Evol. **36**, 220-238. (10.1093/molbev/msy224) 30517664 PMC6367976

[85] Moreno-Estrada A et al. 2014 The genetics of Mexico recapitulates Native American substructure and affects biomedical traits. Science **344**, 1280-1285. (10.1126/science.1251688) 24926019 PMC4156478

[86] Moreno-Estrada A et al. 2013 Reconstructing the population genetic history of the Caribbean. PLoS Genet. **9**, e1003925. (10.1371/journal.pgen.1003925) 24244192 PMC3828151

[87] Homburger JR et al. 2015 Genomic insights into the ancestry and demographic history of South America. PLoS Genet. **11**, e1005602. (10.1371/journal.pgen.1005602) 26636962 PMC4670080

[88] Browning SR, Browning BL, Daviglus ML, Durazo-Arvizu RA, Schneiderman N, Kaplan RC, Laurie CC. 2018 Ancestry-specific recent effective population size in the Americas. PLoS Genet. **14**, e1007385. (10.1371/journal.pgen.1007385) 29795556 PMC5967706

[89] Campbell CL et al. 2012 North African Jewish and non-Jewish populations form distinctive, orthogonal clusters. Proc. Natl Acad. Sci. USA **109**, 13 865-13 870. (10.1073/pnas.1204840109) PMC3427049 22869716

[90] Mooney JA et al. 2018 Understanding the hidden complexity of Latin American population isolates. Am. J. Hum. Genet. **103**, 707-726. (10.1016/j.ajhg.2018.09.013) 30401458 PMC6218714

[91] Goldberg A, Uricchio LH, Rosenberg NA. 2018 Natural Selection in Human Populations. Oxford Bibliographies Online Datasets. (10.1093/obo/9780199941728-0112)

[92] Lachance J, Tishkoff SA. 2013 Population genomics of human adaptation. Annu. Rev. Ecol. Evol. Syst. **44**, 123-143. (10.1146/annurev-ecolsys-110512-135833) 25383060 PMC4221232

[93] Sabeti PC et al. 2006 Positive natural selection in the human lineage. Science **312**, 1614-1620. (10.1126/science.1124309) 16778047

[94] Nielsen R. 2005 Molecular signatures of natural selection. Annu. Rev. Genet. **39**, 197-218. (10.1146/annurev.genet.39.073003.112420) 16285858

[95] Lohmueller KE, Bustamante CD, Clark AG. 2010 The effect of recent admixture on inference of ancient human population history. Genetics **185**, 611-622. (10.1534/genetics.109.113761) 20382834 PMC2881141

[96] Lohmueller KE, Bustamante CD, Clark AG. 2011 Detecting directional selection in the presence of recent admixture in African-Americans. Genetics **187**, 823-835. (10.1534/genetics.110.122739) 21196524 PMC3063676

[97] Racimo F, Sankararaman S, Nielsen R, Huerta-Sánchez E. 2015 Evidence for archaic adaptive introgression in humans. Nat. Rev. Genet. **16**, 359-371. (10.1038/nrg3936) 25963373 PMC4478293

[98] Souilmi Y et al. 2021 Admixture has obscured signals of historical hard sweeps in humans. bioRxiv. **021006**. (10.1101/2020.04.01.021006) PMC9715430 36316412

[99] Busby G, Christ R, Band G, Leffler E, Le QS, Rockett K, Kwiatkowski D, Spencer C. 2017 Inferring adaptive gene-flow in recent African history. bioRxiv. 205252. (10.1101/205252)

[100] Hamid I, Korunes KL, Beleza S, Goldberg A. 2021 Rapid adaptation to malaria facilitated by admixture in the human population of Cabo Verde. Elife **10**, e63177. (10.7554/eLife.63177) 33393457 PMC7815310

[101] Espinoza SC, Laval G, Quintana-Murci L, Patin E. 2022 The genomic signatures of natural selection in admixed human populations. Am. J. Hum. Genet. (10.1016/j.ajhg.2022.02.011) PMC9069077 35259336

[102] Yelmen B, Marnetto D, Molinaro L, Flores R, Mondal M, Pagani L. 2021 Improving selection detection with population branch statistic on admixed populations. Genome Biol. Evol. **13**, evab039. (10.1093/gbe/evab039) 33638983 PMC8046333

[103] Lewontin RC, Krakauer J. 1973 distribution of gene frequency as a test of the theory of the selective neutrality of polymorphisms. Genetics **74**, 175-195. (10.1093/genetics/74.1.175) 4711903 PMC1212935

[104] Robertson A. 1975 Letters to the editors: remarks on the Lewontin-Krakauer test. Genetics **80**, 396. (10.1093/genetics/80.2.396) 1132691 PMC1213336

[105] Nei M, Maruyama T. 1975 Letters to the editors: Lewontin-Krakauer test for neutral genes. Genetics **80**, 395. (10.1093/genetics/80.2.395) 1132690 PMC1213335

[106] Robertson A. 1975 Gene frequency distributions as a test of selective neutrality. Genetics **81**, 775-785. (10.1093/genetics/81.4.775) 1213275 PMC1213434

[107] Beaumont MA. 2005 Adaptation and speciation: what can *F*_st_ tell us? Trends Ecol. Evol. **20**, 435-440. (10.1016/j.tree.2005.05.017) 16701414

[108] Tang H, Choudhry S, Mei R, Morgan M, Rodriguez-Cintron W, Burchard EG, Risch NJ. 2007 Recent genetic selection in the ancestral admixture of Puerto Ricans. Am. J. Hum. Genet. **81**, 626-633. (10.1086/520769) 17701908 PMC1950843

[109] Cheng JY, Stern AJ, Racimo F, Nielsen R. 2021 Detecting selection in multiple populations by modeling ancestral admixture components. Mol. Biol. Evol. **39**, msab294. (10.1093/molbev/msab294) PMC8763095 34626111

[110] Jin W, Xu S, Wang H, Yu Y, Shen Y, Wu B, Jin L. 2012 Genome-wide detection of natural selection in African Americans pre- and post-admixture. Genome Res. **22**, 519-527. (10.1101/gr.124784.111) 22128132 PMC3290787

[111] Patin E et al. 2017 Dispersals and genetic adaptation of Bantu-speaking populations in Africa and North America. Science **356**, 543-546. (10.1126/science.aal1988) 28473590

[112] Fernandes V, Brucato N, Ferreira JC, Pedro N, Cavadas B, Ricaut F-X, Alshamali F, Pereira L. 2019 Genome-wide characterization of Arabian Peninsula populations: shedding light on the history of a fundamental bridge between continents. Mol. Biol. Evol. **36**, 575-586. (10.1093/molbev/msz005) 30649405

[113] Bhatia G et al. 2014 Genome-wide scan of 29,141 African Americans finds no evidence of directional selection since admixture. Am. J. Hum. Genet. **95**, 437-444. (10.1016/j.ajhg.2014.08.011) 25242497 PMC4185117

[114] Haller BC, Messer PW. 2019 SLiM 3: forward genetic simulations beyond the Wright–Fisher model. Mol. Biol. Evol. **36**, 632-637. (10.1093/molbev/msy228) 30517680 PMC6389312

[115] Vicuña L, Klimenkova O, Norambuena T, Martinez FI, Fernandez MI, Shchur V, Eyheramendy S. 2020 Postadmixture selection on Chileans targets haplotype involved in pigmentation, thermogenesis and immune defense against pathogens. Genome Biol. Evol. **12**, 1459-1470. (10.1093/gbe/evaa136) 32614437 PMC7487163

[116] Svedberg J, Shchur V, Reinman S, Nielsen R, Corbett-Detig R. 2021 Inferring adaptive introgression using hidden Markov models. Mol. Biol. Evol. **38**, 2152-2165. (10.1093/molbev/msab014) 33502512 PMC8097282

[117] Hodgson JA, Pickrell JK, Pearson LN, Quillen EE, Prista A, Rocha J, Soodyall H, Shriver MD, Perry GH. 2014 Natural selection for the Duffy-null allele in the recently admixed people of Madagascar. Proc. R. Soc. B **281**, 20140930. (10.1098/rspb.2014.0930) PMC4100512 24990677

[118] Triska P, Soares P, Patin E, Fernandes V, Cerny V, Pereira L. 2015 Extensive admixture and selective pressure across the Sahel Belt. Genome Biol. Evol. **7**, 3484-3495. (10.1093/gbe/evv236) 26614524 PMC4700964

[119] Pierron D et al. 2018 Strong selection during the last millennium for African ancestry in the admixed population of Madagascar. Nat. Commun. **9**, 932. (10.1038/s41467-018-03342-5) 29500350 PMC5834599

[120] Shchur V, Svedberg J, Medina P, Corbett-Detig R, Nielsen R. 2020 On the distribution of tract lengths during adaptive introgression. G3 **10**, 3663-3673. (10.1534/g3.120.401616) 32763953 PMC7534438

[121] Peterson RE et al. 2019 Genome-wide association studies in ancestrally diverse populations: opportunities, methods, pitfalls, and recommendations. Cell **179**, 589-603. (10.1016/j.cell.2019.08.051) 31607513 PMC6939869

[122] Tian C, Gregersen PK, Seldin MF. 2008 Accounting for ancestry: population substructure and genome-wide association studies. Hum. Mol. Genet. **17**, R143-R150. (10.1093/hmg/ddn268) 18852203 PMC2782357

[123] Sul JH, Martin LS, Eskin E. 2018 Population structure in genetic studies: confounding factors and mixed models. PLoS Genet. **14**, e1007309. (10.1371/journal.pgen.1007309) 30589851 PMC6307707

[124] Martin AR, Gignoux CR, Walters RK, Wojcik GL, Neale BM, Gravel S, Daly MJ, Bustamante CD, Kenny EE. 2017 Human demographic history impacts genetic risk prediction across diverse populations. Am. J. Hum. Genet. **100**, 635-649. (10.1016/j.ajhg.2017.03.004) 28366442 PMC5384097

[125] Wojcik GL et al. 2019 Genetic analyses of diverse populations improves discovery for complex traits. Nature **570**, 514-518. (10.1038/s41586-019-1310-4) 31217584 PMC6785182

[126] Duncan L, Shen H, Gelaye B, Meijsen J, Ressler K, Feldman M, Peterson R, Domingue B. 2019 Analysis of polygenic risk score usage and performance in diverse human populations. Nat. Commun. **10**, 3328. (10.1038/s41467-019-11112-0) 31346163 PMC6658471

[127] Majara L, Kalungi A, Koen N, Zar H, Stein DJ, Kinyanda E, Atkinson EG, Martin AR.. 2021 Low generalizability of polygenic scores in African populations due to genetic and environmental diversity. bioRxiv, 426453. (10.1101/2021.01.12.426453)

[128] Bitarello BD, Mathieson I. 2020 Polygenic scores for height in admixed populations. G3 **10**, 4027-4036. (10.1534/g3.120.401658) 32878958 PMC7642950

[129] Cavazos TB, Witte JS. 2021 Inclusion of variants discovered from diverse populations improves polygenic risk score transferability. HGG Adv **2**, 100017. (10.1016/j.xhgg.2020.100017) 33564748 PMC7869832

[130] Mills MC, Rahal C. 2019 A scientometric review of genome-wide association studies. Commun. Biol. **2**, 9. (10.1038/s42003-018-0261-x) 30623105 PMC6323052

[131] Fish AE, Crawford DC, Capra JA, Bush WS. 2018 Local ancestry transitions modify SNP-trait associations. Pac. Symp. Biocomput. **2018**, 424-435. (10.1142/9789813235533_0039) PMC5728664 29218902

[132] Novembre J, Barton NH. 2018 Tread lightly interpreting polygenic tests of selection. Genetics **208**, 1351-1355. (10.1534/genetics.118.300786) 29618592 PMC5886544

[133] Wang Y, Guo J, Ni G, Yang J, Visscher PM, Yengo L. 2020 Theoretical and empirical quantification of the accuracy of polygenic scores in ancestry divergent populations. Nat. Commun. **11**, 3865. (10.1038/s41467-020-17719-y) 32737319 PMC7395791

[134] Guan Y. 2014 Detecting structure of haplotypes and local ancestry. Genetics **196**, 625-642. (10.1534/genetics.113.160697) 24388880 PMC3948796

[135] Zhong Y, Perera MA, Gamazon ER. 2019 On using local ancestry to characterize the genetic architecture of human traits: genetic regulation of gene expression in multiethnic or admixed populations. Am. J. Hum. Genet. **104**, 1097-1115. (10.1016/j.ajhg.2019.04.009) 31104770 PMC6562007

